# Association of mortality and aspirin prescription for COVID-19 patients at the Veterans Health Administration

**DOI:** 10.1371/journal.pone.0246825

**Published:** 2021-02-11

**Authors:** Thomas F. Osborne, Zachary P. Veigulis, David M. Arreola, Satish M. Mahajan, Eliane Röösli, Catherine M. Curtin

**Affiliations:** 1 US Department of Veterans Affairs, Palo Alto Healthcare System, Palo Alto, California, United States of America; 2 Department of Radiology, Stanford University School of Medicine, Stanford, California, United States of America; 3 US Department of Veterans Affairs, Central Iowa Health Care System, Des Moines, Iowa, United States of America; 4 Department of Medicine, Stanford University School of Medicine, Stanford, California, United States of America; 5 Department of Surgery, Stanford University School of Medicine, Stanford, California, United States of America; Erasmus Medical Center, NETHERLANDS

## Abstract

There is growing evidence that thrombotic and inflammatory pathways contribute to the severity of COVID-19. Common medications such as aspirin, that mitigate these pathways, may decrease COVID-19 mortality. This retrospective assessment was designed to quantify the correlation between pre-diagnosis aspirin and mortality for COVID-19 positive patients in our care. Data from the Veterans Health Administration national electronic health record database was utilized for the evaluation. Veterans from across the country with a first positive COVID-19 polymerase chain reaction lab result were included in the evaluation which comprised 35,370 patients from March 2, 2020 to September 13, 2020 for the 14-day mortality cohort and 32,836 patients from March 2, 2020 to August 28, 2020 for the 30-day mortality cohort. Patients were matched via propensity scores and the odds of mortality were then compared. Among COVID-19 positive Veterans, preexisting aspirin prescription was associated with a statistically and clinically significant decrease in overall mortality at 14-days (OR 0.38, 95% CI 0.32–0.46) and at 30-days (OR 0.38, 95% CI 0.33–0.45), cutting the odds of mortality by more than half. Findings demonstrated that pre-diagnosis aspirin prescription was strongly associated with decreased mortality rates for Veterans diagnosed with COVID-19. Prospective evaluation is required to more completely assess this correlation and its implications for patient care.

## Introduction

Veterans Health Administration (VA) is the largest integrated healthcare system in the United States (U.S.) and enrolled Veterans represent a population at increased risk of poor COVID-19 outcomes due to older age and multiple comorbidities [1–5]. As part of our continual quality improvement and assessment efforts, we have been developing and validating predictive models to optimize care strategies. Our recent work demonstrated that existing electronic health record (EHR) data can be utilized at VA to assess risk in the form of the Care Assessment Needs (CAN) score for different groups of Veterans including those battling COVID-19 [6, 7]. In the process of optimizing COVID-19 specific predictive models, we identified a strong correlation between preexisting aspirin prescription and decreased all-cause mortality. Given the urgency of this crisis, and the potential for drug repurposing to improve outcomes, this analysis is our in-depth evaluation of the correlation between standard care aspirin prescription and mortality for COVID-19 positive Veterans.

## Materials and methods

### Design

Data was obtained from the VA national Corporate Data Warehouse (CDW), a central relational database repository that aggregates EHR records from 1,255 VA health care facilities across the U.S. This database includes enrolled Veterans as well as a very small number of non-Veterans such as qualified spouses. Our work to refine VA risk assessment tools for COVID-19 outcomes started with binary logistic regression modeling of mortality on common variables available in our EHR such as comorbidities and pre-diagnosis medications. A specific medication or medication class was included in the assessment if utilized by more than 10% of the cohort population. This resulted in the inclusion of 17 specific medications and 14 medication classes, which are listed in the S1 Table. There was a strong correlation between pre-diagnosis standard care use of aspirin and decreased all-cause mortality in each of the logistic regression models we evaluated. We utilized log odds of at least -0.05 in mortality as an inclusion criterion for medications in the assessment, and of the 31 medications and medication classes evaluated, pre-diagnosis aspirin prescription was the only one that met this criterion (mortality decreased by 36% with a log odds of -0.45). Retrospective analysis was then performed on both aspirin and non-aspirin groups for 14-day and 30-day mortality assessment. The Care Assessment Needs (CAN) score, the Charlson Comorbidity Index (CCI), specific comorbidities, and demographics were utilized to compare baseline characteristics of the different cohorts.

### Patients

CDW lab tables were queried to identify the first positive COVID-19 polymerase chain reaction (PCR) results for patients. For this evaluation, the time of the first positive COVID-19 test administration was considered the time of diagnosis. Test results performed outside of VA were not included in the evaluation to reduce potential bias due to test variability and incomplete access to care records. Patients without a calculated CAN score within 6 months prior to their first positive COVID-19 lab results were excluded since a patient’s CAN score was utilized to inform current health status and patients not actively utilizing VA care will not have a CAN score calculated.

### Variables

The aspirin cohort was defined as those patients who had delivery of an active aspirin prescription by the VA pharmacy at the time of a positive COVID-19 lab test. If a patient had no refills at the time of diagnosis, then the prescription was only considered active if it was filled up to 30 days prior to the positive COVID-19 lab test. Patients who had non-VA aspirin prescriptions documented as an active medication in our EHR, were also considered active, and were included in the treatment group. All other COVID-19 patients, with no documented active aspirin prescription, were assigned to the control cohort. This methodology was adopted based on previous work utilizing VA EHR data to assess aspirin use [8].

Patients’ health risk was controlled for by using the VA CAN score. The CAN score is a tool that assesses patients’ risk of morbidity and mortality using a wide array of data available in the EHR, including socio-demographics, clinical diagnoses, vital signs, medications, lab values, and health care utilization data (S2 Table). Our previous work has established that the CAN score successfully risk-stratifies COVID-19 patient outcomes with the strongest performance demonstrated in predicting mortality [6, 7]. The CAN score ranges from 0 to 99, with a higher score representing a greater risk patient. We utilized the CAN 1-year mortality model (version 2.5), which is automatically computed weekly based on all living Veterans who actively receive care services from VA.

The primary outcomes for this assessment are 14-day and 30-day all-cause mortality within or outside of hospital care. Mortality is identified by date and time of death listed in CDW. The time windows for the evaluation start on the date of the first positive COVID-19 test recorded at VA. The 14-day and 30-day cohort have different end dates to allow for appropriate follow-up time window to elapse before analysis could be performed for all-cause mortality.

### Statistical analysis

For both the 14-day and 30-day mortality outcomes that were evaluated, unadjusted odds ratios using contingency tables were first computed to quantify the difference in mortality between the aspirin treatment and control groups. Given the retrospective observational nature of the data, leading to differences in the treatment and control groups, propensity score matching was applied to reduce confounding effects. To do so, the treatment and control group were matched one-to-one on the unscaled covariates of age, gender, and CAN score (1-year mortality) with the RStudio “MatchIt” library (V3.6.2) using the commonly used caliper setting of 0.1. Race (White vs. other) was not included as a covariate in the matching algorithm since our assessment demonstrated that it was not significantly associated with the treatment assignment nor the outcome. New contingency tables and adjusted odds ratios were then computed on the matched cohort for both the all-cause 14-day and 30-day mortality outcomes. Fixing the control cohort as the reference group, odds ratios are reported for the aspirin treatment cohort as point estimates with 95% confidence intervals.

This quality improvement and assessment project received a Determination of Non-Research from Stanford IRB (Stanford University, Stanford, CA, USA), as well as by VA determination.

## Results

### Patient characteristics

**Fig 1:**
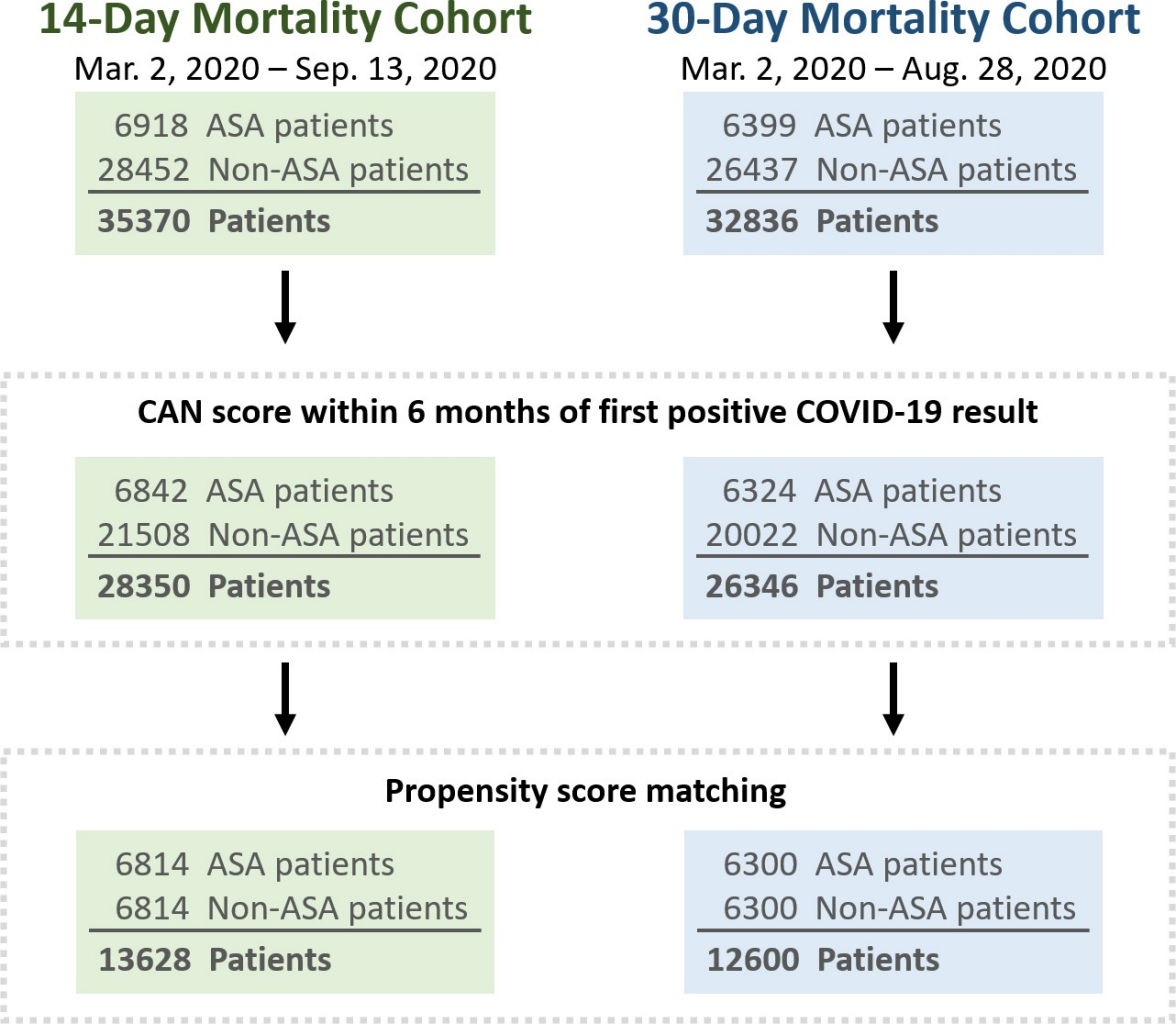
Cohorts for the 14-day and 30-day mortality evaluation of Veterans diagnosed with COVID-19. ASA = Aspirin.

The initial cohort of COVID-19 positive Veterans included 35,370 patients identified from March 2, 2020 to September 13, 2020 for the 14-day mortality evaluation, and 32,836 patients identified from March 2, 2020 to August 28, 2020 for the 30-day mortality evaluation. Patients without a CAN score calculated within six months of the first positive COVID-19 PCR result were excluded leaving 28,350 patients in the 14-day mortality cohort, and 26,346 in the 30-day mortality cohort (Fig 1).

In both cohorts, approximately 24% of patients were prescribed aspirin prior to COVID-19 diagnosis. The majority of both the unmatched cohorts are male, which is expected for our VA population. Both cohorts were virtually the same age (58 years each) and in the same health as represented by the CAN score (CAN of 44 each) and Charlson Comorbidity Index (CCI of 2.7 each). The similarity of the 14-day and 30-day cohorts is expected as all subjects in the 30-day group were also part of the 14-day group. More than half of all patients had hypertension, more than 30% had chronic pulmonary disease and diabetes, and 15% had congestive heart failure (Table 1).

**Table 1:** Characteristics of the 14-day and the 30-day mortality assessment cohorts.

	14-Day Mortality n = 28350	30-Day Mortality n = 26346
	n (%)	n (%)
**Follow-up Period Mortality**	956 (3.37)	1531 (5.81)
	**Mean (SD)**	**Mean (SD)**
**Patient Age in Years**	58.44 (16.68)	58.41 (16.70)
**Charlson Comorbidity Index**^**8**^	2.66 (3.12)	2.66 (3.13)
**Care Assessment Need (CAN) 1 Year Mort.**	44.43 (31.85)	44.45 (31.89)
	**n (%)**	**n (%)**
**Male**	25276 (89.2)	23487 (89.1)
**Hypertension**	17561 (61.9)	16308 (61.9)
**Chronic Pulmonary Disease**	9149 (32.3)	8480 (32.2)
**Congestive Heart Failure**	4113 (14.5)	3851 (14.6)
**Diabetes**	10353 (36.5)	9619 (36.5)
**American Indian or Alaska Native**	339 (1.2)	302 (1.2)
**Asian**	337 (1.2)	312 (1.2)
**Black or African American**	9883 (35.5)	9374 (36.2)
**Native American or other Pacific Islander**	300 (1.1)	276 (1.1)
**White**	15237 (53.7)	13997 (53.1)
**Race not reported**	2254 (8.0)	2085 (7.9)

### Treatment and control groups

**Fig 2:**
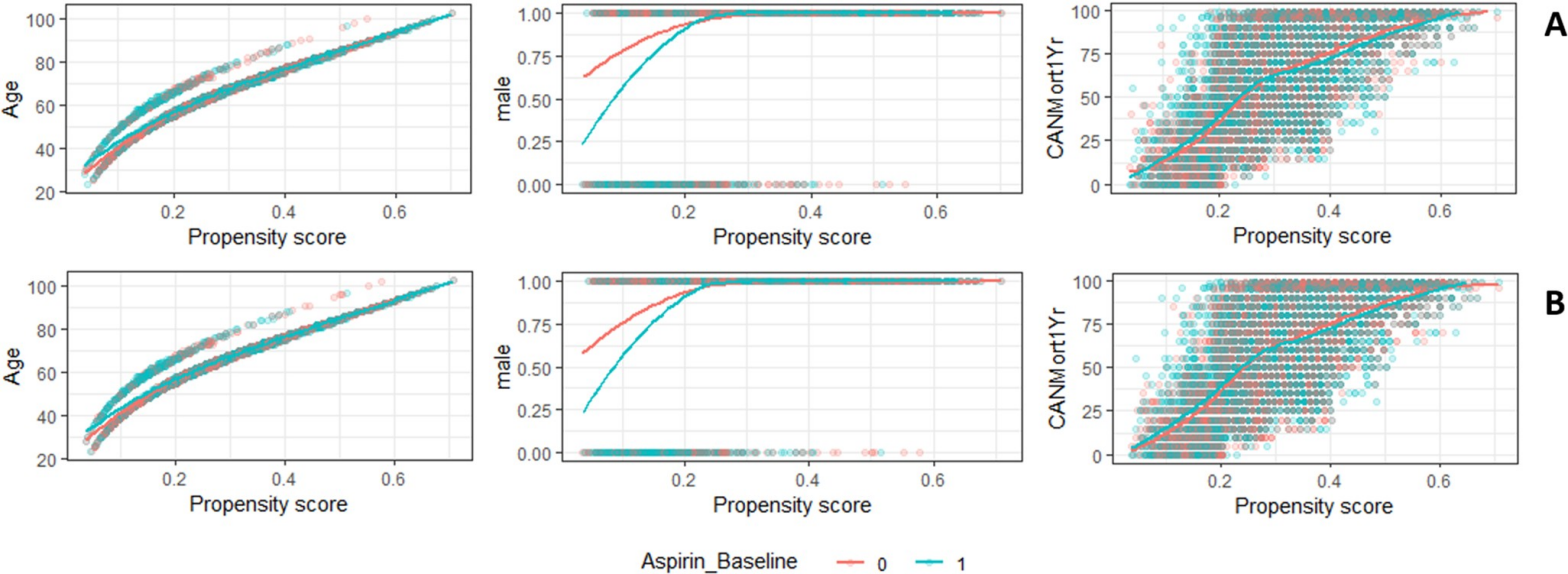
Scatter plots with fitted lines of the association between the propensity score and the matched covariates (age, gender and CAN 1-year mortality score). 30-day mortality assessment cohort (A) and the 14-day mortality assessment cohort (B).

For the treatment and control groups, the differences in age, gender, and CAN score were resolved after applying propensity score matching (Tables 2 and 3). The scatter plots of the propensity score as a function of the covariates age, gender, and CAN score demonstrate very closely fitting curves to further confirm this assessment (Fig 2).

**Table 2:** Patient characteristics for the 14-day mortality assessment cohort before and after propensity score matching.

	Complete Cohort		Propensity Score Matched Cohort	
	No Aspirin n = 21508 n (%)	Aspirin n = 6842 n (%)	No Aspirin n = 6814 n (%)	Aspirin n = 6814 n (%)
**14-Day Mortality**	785 (3.65)	171 (2.50)	427 (6.27)	170 (2.49)
	**Mean (SD)**	**Mean (SD)**	**Mean (SD)**	**Mean (SD)**
**Age Years**	55.54 (17.17)	67.55 (10.79)	67.36 (11.11)	67.50 (10.76)
**Charlson Comorbidity Index**	2.16 (2.90)	4.25 (3.28)	3.34 (3.30)	4.25 (3.28)
**CAN 1 Year Mortality**	39.92 (31.78)	58.59 (27.60)	58.80 (28.85)	58.63 (27.61)
	**n (%)**	**n (%)**	**n (%)**	**n (%)**
**Male**	18755 (87.2)	6521 (95.3)	6584 (96.6)	6493 (95.3)
**Hypertension**	11446 (53.2)	6115 (89.4)	4962 (72.8)	6089 (89.4)
**Chronic Pulmonary Disease**	6145 (28.6)	3004 (43.9)	2579 (37.8)	2999 (44.0)
**Congestive Heart Failure**	2323 (10.8)	1790 (26.2)	1237 (18.2)	1785 (26.2)
**Diabetes**	6085 (28.3)	4268 (62.4)	2841 (41.7)	4256 (62.5)
**American Indian or Alaska Native**	271 (1.3)	68 (1.0)	71 (1.0)	68 (1.0)
**Asian**	291 (1.4)	46 (0.7)	61 (0.9)	46 (0.7)
**Black or African American**	7399 (35.1)	2484 (36.6)	2335 (34.3)	2474 (36.3)
**Native American or other Pacific Islander**	237 (1.1)	63 (0.9)	61 (0.9)	63 (0.9)
**White**	11478 (53.4)	3759 (54.0)	3792 (55.7)	3745 (55.0)
**Race not reported**	1832 (8.5)	422 (6.2)	494 (7.2)	418 (6.1)

**Table 3:** Patient characteristics for the 30-day mortality assessment cohort before and after propensity score matching.

	Complete Cohort		Propensity Score Matched Cohort	
	No Aspirin n = 20022 n (%)	Aspirin n = 6324 n (%)	No Aspirin n = 6300 n (%)	Aspirin n = 6300 n (%)
**30-Day Mortality**	1257 (6.28)	274 (4.33)	661 (10.49)	272 (4.32)
	**Mean (SD)**	**Mean (SD)**	**Mean (SD)**	**Mean (SD)**
**Age Years**	55.55 (17.21)	67.46 (10.82)	67.27 (11.15)	67.41 (10.77)
**Charlson Comorbidity Index**	2.16 (2.91)	4.23 (3.27)	3.30 (3.26)	4.23 (3.27)
**CAN 1 Year Mortality**	40.01 (31.86)	58.50 (27.64)	58.65 (28.98)	58.41 (27.66)
	**n (%)**	**n (%)**	**n (%)**	**n (%)**
**Male**	17466 (87.2)	6021 (95.2)	6083 (96.6)	5997 (95.2)
**Hypertension**	10653 (53.2)	5655 (89.4)	4574 (72.6)	5634 (89.4)
**Chronic Pulmonary Disease**	5710 (28.5)	2770 (43.8)	2388 (37.9)	2758 (43.8)
**Congestive Heart Failure**	2184 (10.9)	1667 (26.4)	1163 (18.5)	1655 (26.3)
**Diabetes**	5670 (28.3)	3949 (62.4)	2599 (41.3)	3936 (62.5)
**American Indian or Alaska Native**	242 (1.2)	60 (1.0)	62 (1.0)	60 (1.0)
**Asian**	268 (1.4)	44 (0.7)	59 (0.9)	44 (0.7)
**Black or African American**	7020 (35.8)	2354 (37.5)	2252 (35.7)	2348 (37.3)
**Native American or other Pacific Islander**	222 (1.1)	54 (0.9)	55 (0.9)	54 (0.9)
**White**	10576 (52.8)	3421 (54.1)	3435 (54.5)	3407 (54.1)
**Race not reported**	1694 (8.5)	391 (6.2)	437 (6.9)	387 (6.1)

### Mortality

COVID-19 positive VA patients with active aspirin prescriptions have a significantly decreased risk of mortality as indicated by unadjusted odds ratios of 0.68 (95% CI of 0.57–0.80) at 14 days, and 0.68 (95% CI of 0.59–0.77) at 30 days after diagnosis. Accounting for age, gender, and CAN score via propensity score matching, the adjusted odds of dying while having an aspirin prescription was 0.38 (95% CI of 0.32–0.46) at 14-days, and 0.38 (95% CI of 0.33–0.45) at 30-days (Table 4). In the propensity-matched cohort, these adjusted odds ratios represent a drop in 14-day mortality from 6.3% (control group) to 2.5% (treatment group) and a drop in 30-day mortality from 10.5% (control group) to 4.3% (treatment group).

**Table 4:** Unadjusted and adjusted odds ratios for the 14-day and 30-day mortality aspirin cohorts (95% confidence interval). All odds ratio p-values are < 0.001.

	14-Day Mortality	30-Day Mortality
**Unadjusted OR**	0.68 (0.57–0.80)	0.68 (0.59–0.77)
**Adjusted OR**	0.38 (0.32–0.46)	0.38 (0.33–0.45)

## Discussion

As part of our COVID-19 quality improvement and assessment efforts at VA, we have been internally developing and validating COVID-19 prediction models to optimize Veteran care. In the process of assessing logistic regression models, we discovered a strong correlation between preexisting aspirin prescription and decreased mortality in COVID-19 positive Veterans. This observation also confirms a recent study of 400 patients, which found aspirin use to be associated with lower COVID-19 mortality [9]. Our large national study highlights that aspirin is an important target for additional assessment in the treatment of COVID-19.

The impact of propensity score matching on the odds ratio points to a strong confounding of aspirin’s treatment effect by the covariates age, gender, and CAN score. Accounting for these risk factors that are commonly associated with aspirin prescriptions, findings demonstrated that baseline aspirin prescriptions imparted a strong decrease in mortality, essentially cutting the risk of an adverse outcome by more than half in our population. In addition, these results are statistically and clinically significant for mortality outcomes measured in both the 14-day and 30-day timeframes.

The associated benefit of aspirin on Veterans in this disease may be related to several factors. A leading possibility is that aspirin’s systemic antithrombotic effects could disrupt the increasingly recognized life-threatening risk of thrombotic events related to COVID-19. As an example, a recent study of autopsy findings in twelve COVID-19 patients found 58% had deep vein thrombosis and 33% had pulmonary embolus as the direct cause of death [10]. There is also an increased rate of acute ischemic strokes in COVID-19 patients, which is felt to be a consequence of the hypercoagulable state associated with the infection [11, 12]. Arterial thrombosis has also been characterized in critically ill COVID-19 patients, resulting in loss of limbs and life [13]. Notably, this evaluation presents positive associations for a population of patients who were on aspirin at the time of diagnosis with COVID-19, and not as a new treatment after becoming acutely ill. This may be a crucial distinction because aspirin inhibits platelets as well as inflammatory cytokines that lead to pathologic platelet aggregation. These effects of aspirin can prevent clot formation but not thrombolysis of an existing clot [14, 15]. Therefore, if COVID-19 hypercoagulability induced thrombotic events, such as myocardial infarction, pulmonary embolism, limb ischemia, and stroke, are an important cause of acute decompensation, the presence of the antiplatelet effects of aspirin before COVID-19 infection could be protective. In addition, since our population may be at higher baseline risk for thrombotic events due to older age and comorbidities, the superimposed impact of a COVID-19 induced prothrombotic state may have disproportionate consequences for our patients that is important to understand as we continually work to rapidly optimize care. Future study will also be needed to assess how aspirin fits into this hypercoagulable state as aspirin has been considered most effective in prevention of arterial thrombotic diseases and less so for venous thrombotic events. Likewise, the anti-inflammatory effect of aspirin may also have an independent or synergistic beneficial effect. There are other potential pathways to consider in which aspirin could positively impact patients with COVID-19 such as inhibiting virus replication [16].

There are cautions to be considered when discussing aspirin use in the setting of COVID-19. Early in this pandemic, there was concern that NSAIDs could be related to adverse cardiovascular and pulmonary outcomes and there was an editorial published that suggested potential harm with NSAID prescription for COVID-19 patients [17]. However, the World Health Organization subsequently published a brief report stating that: “…there is no evidence of severe adverse events, acute health care utilization, long-term survival, or quality of life in patients with COVID-19, as a result of the use of NSAID” [18]. Nevertheless, there are many aspirin contraindications to take into consideration such as in the setting of disseminated intravascular coagulation and other bleeding disorders, which can result in uncontrolled hemorrhage, as well as in children due to the risk of Reye’s syndrome.

There are critical strengths of this assessment; our data source included a large number of patients, spanning a diverse geographic range, from an integrated longitudinal EHR database. In addition, because VA practices purposeful adverse patient selection, many Veterans rely on VA for over the counter (OTC) medications. Therefore, our database offers a broader scope of care and a more complete record of outpatient aspirin use, which is a crucial variable. However, there are also Veterans with better financial means, who may not rely on VA for OTC medications, and it is possible that some of these patients may not have reported their aspirin use to their clinicians even though medication reconciliation is a national VA system-wide policy for clinical visits and the VA EHR database is designed to include non-VA prescribed OTC medications. Since those with better financial means tend to have better healthcare outcomes, this could result in an underestimation of the beneficial association of aspirin [19, 20]. The relative recent emergence of this pandemic highlights an important limitation of all COVID-19 assessments such as reporting of mortality occurring outside of care facilities, which may be delayed and could also underestimate the impact.

There are several areas that deserve additional assessment as more data becomes available. For example, this study did not have information on the dosage of aspirin. Future, sub-cohort analysis of the relative impact of different dosages of aspirin, as well as the effect of less common anticoagulation medications, will become more statistically significant over time as datasets increase in size. Importantly, as a retrospective evaluation, we cannot establish direct cause and effect, only correlation that deserves dedicated controlled trial to assess the potential of aspirin as a drug repurposing option for this population. Finally, when compared to other healthcare systems, the VA population is statistically older with multiple comorbidities, may face unique Veteran related health challenges, and is disproportionally male. As a result, VA specific patient variables, including potential sex differences in biological pathways of clot formation, limit the generalizability of this assessment. However, as more information becomes available, we may be able to gain greater insights about sex related outcome variations in our patients.

## Conclusion

Aspirin prescription was discovered to be strongly associated with decreased mortality rates for COVID-19 positive patients enrolled at VA. Additional prospective evaluation is required to more completely understand this correlation and the potential implications for improving care.

## Supporting information

S1 Table The 31 medication variables utilized in the logistic regression analysis. If a unique medication was present in more than 10% of the cohort, it was included as its own variable. All other medications were grouped as "other" by drug class. This resulted in the inclusion of 31 variables across 14 separate medication classes.
(DOCX)

S2 Table The variables utilized to calculate the CAN 1-year mortality model (version 2.5). (DOCX)
