# Supplementary material for: Association of mortality and aspirin prescription for COVID-19 patients at the Veterans Health Administration
Source: PLoS One. 2021 Feb 11;16(2):e0246825. doi: 10.1371/journal.pone.0246825 (PMC7877611; doi:10.1371/journal.pone.0246825)
Supplement: S1 Table — If a unique medication was present in more than 10% of the cohort, it was included as its own variable. All other medications were grouped as "other" by drug class. This resulted in the inclusion of 31 variables across 14 separate medication classes. (DOCX) [file pone.0246825.s001.docx]

**S1 Table.** The 31 medication variables utilized in the logistic regression analysis. If a unique medication was present in more than 10% of the cohort, it was included as its own variable. All other medications were grouped as "other" by drug class. This resulted in the inclusion of 31 variables across 14 separate medication classes.
