# Supplementary material for: Association of mortality and aspirin prescription for COVID-19 patients at the Veterans Health Administration
Source: PLoS One. 2021 Feb 11;16(2):e0246825. doi: 10.1371/journal.pone.0246825 (PMC7877611; doi:10.1371/journal.pone.0246825)
Supplement: S2 Table — (DOCX) [file pone.0246825.s002.docx]

**S2 Table.** Variables utilized to calculate the CAN 1-year mortality model (version 2.5)
